# Integrated Metabolomic and Transcriptomic Analysis to Characterize Cutin Biosynthesis between Low- and High-Cutin Genotypes of *Capsicum chinense* Jacq

**DOI:** 10.3390/ijms21041397

**Published:** 2020-02-19

**Authors:** Purushothaman Natarajan, Tolulope Abodunrin Akinmoju, Padma Nimmakayala, Carlos Lopez-Ortiz, Marleny Garcia-Lozano, Benjamin J. Thompson, John Stommel, Umesh K. Reddy

**Affiliations:** 1 Department of Biology and Gus R. Douglass Institute, West Virginia State University, Institute, WV 25112, USA; pnatarajan@wvstateu.edu (P.N.); takinmoju@wvstateu.edu (T.A.A.); carlos.ortiz@wvstateu.edu (C.L.-O.); mgarcialozano@wvstateu.edu (M.G.-L.); thompson504@live.marshall.edu (B.J.T.); 2 Department of Genetic Engineering, SRM Institute of Science and Technology, Chennai 603203, TN, India; 3 Genetic Improvement of Fruits and Vegetables Laboratory, U.S. Department of Agriculture, Agricultural Research Service, Beltsville Agricultural Research Center, Beltsville, MD 20705-2325, USA; john.stommel@usda.gov

**Keywords:** GDSL lipase, GPAT6, cutin, habaneros, *Capsicum chinense*, fruit, RNA-Seq

## Abstract

Habanero peppers constantly face biotic and abiotic stresses such as pathogen/pest infections, extreme temperature, drought and UV radiation. In addition, the fruit cutin lipid composition plays an important role in post-harvest water loss rates, which in turn causes shriveling and reduced fruit quality and storage. In this study, we integrated metabolome and transcriptome profiling pertaining to cutin in two habanero genotypes: PI 224448 and PI 257145. The fruits were selected by the waxy or glossy phenotype on their surfaces. Metabolomics analysis showed a significant variation in cutin composition, with about 6-fold higher cutin in PI 257145 than PI 224448. It also revealed that 10,16-dihydroxy hexadecanoic acid is the most abundant monomer in PI 257145. Transcriptomic analysis of high-cutin PI 257145 and low-cutin PI 224448 resulted in the identification of 2703 statistically significant differentially expressed genes, including 1693 genes upregulated and 1010 downregulated in high-cutin PI 257145. Genes and transcription factors such as GDSL lipase, glycerol-3 phosphate acyltransferase 6, long-chain acyltransferase 2, cytochrome P450 86A/77A, SHN1, ANL2 and HDG1 highly contributed to the high cutin content in PI 257145. We predicted a putative cutin biosynthetic pathway for habanero peppers based on deep transcriptome analysis. This is the first study of the transcriptome and metabolome pertaining to cutin in habanero peppers. These analyses improve our knowledge of the molecular mechanisms regulating the accumulation of cutin in habanero pepper fruits. These resources can be built on for developing cultivars with high cutin content that show resistance to biotic and abiotic stresses with superior postharvest appearance.

## 1. Introduction

Hot peppers (*Capsicum chinense* Jacq.), popularly known as habaneros, are domesticated from tropical regions of Central America and have great economic significance in terms of culinary, pharmaceutical and ornamental perspectives. Their fruits are a good source of vitamins, antioxidants and other phytonutrients, including major important alkaloids such as phenolics, carotenoids, flavonoids and capsaicinoids [1,2]. Habanero pepper fruits are subjected to desiccation and postharvest wilting because of their hollow shape and limited water-holding capacity. The abundance of cuticle in the pericarp can resist desiccation, but pepper cuticle development is not yet well understood [3,4]. Cuticle is known to play a critical role in plant survival because its primary physiological function is as a sealant around plant tissues to protect against drought conditions and prevent desiccation by reducing nonstomatal water loss [5,6,7]. The cuticle structure is diverse among different species but is made up of a polyester cutin that is covered with waxes (intracuticular and epicuticular). Cuticle, a hydrophobic polymer synthesized by the epidermal cells, is a major physiological trait acquired by plants during evolution for survival in dehydrated conditions. It also coordinates the interaction between a plant and its environment by limiting UV radiation and mechanical damage and is a defense against pathogen entry. In terms of chemical composition, the cuticle is a polyester matrix of cutin embedded with waxes [8].

Cutin is the major constituent of the plant cuticle and makes up about 80% of the plant cuticle. It is an insoluble, covalently cross-linked polymer that is synthesized by epidermal cells in higher plants [5,6,9]. Cutin is made up of organic chemicals that include glycerol, hydroxylated fatty acids and hydroxylated epoxy compounds with carbon atom chains of lengths 16 and 18 and phenolic compounds [5,10,11,12,13]. Cutin composition and its genetic basis have been studied in model plants such as *Arabidopsis*, tomato and rice [14,15,16,17]. In *Arabidopsis*, several genes including GPAT6, GDSL lipase, LACS, CYP86A, CYP77A, ABCG32 and ABCG11 involved in cutin initiation and development have been identified [7,18,19]. In peppers, Parsons et al. [4] reported that the cuticle of *Capsicum annum* fruit show variations in composition among species and cultivars [4,7,20]. Additionally, cuticle composition varies across pepper cultivars, which in turn affects the response to biotic and abiotic stresses [21]. However, a better understanding of the molecular basis of this monomer composition is important for using cutin for crop improvement in pepper [4].

Recent progress in “omics” approaches is being utilized for tracking the metabolites and genes involved in cutin biosynthesis, transport and regulation in plant tissues [4,19,22]. Owing to the widely proposed significance of cuticle in plant physiology and metabolism, the metabolite profile of cutin has been explored extensively in the model plant *Arabidopsis* and other crops such as barley, tomato, rice and maize [15,17,23]. Different aspects of cuticle biosynthesis have been considered in *Arabidopsis* and tomato fruits [5,12,13], however, there are no studies reported in habanero peppers in terms of whole fruit transcriptome and metabolome to understand cutin accumulation and metabolism. Hence, the current study aimed to understand cutin biosynthesis in habanero peppers by taking advantage of integrated RNA-Seq and metabolome analysis to study cutin biosynthesis in fruit tissues of diverse *C. chinense* genotypes. Here, we used gas chromatography–mass spectrometry (GC-MS) of two different habanero peppers, PI 224448 from Costa Rica and PI 257145 from Peru, for metabolome analysis to study cutin composition across genotypes. We also performed deep paired-end RNA-Seq of the two samples by using the Illumina Nextseq 500 platform to identify differentially expressed genes (DEGs) and pathways associated with cutin and other traits by comparing PI 257145 and PI 224448. This is the first study to generate transcriptome and metabolome data pertaining to cutin in habanero peppers. These results can be used by plant breeders for hot pepper fruit quality improvement via biotechnological modifications and can also serve as a model for the other Solanaceae crops.

## 2. Results and Discussion

### 2.1. Metabolomic Analysis of Cutin Monomers

Raw cutin from two habanero genotypes, PI 224448 and PI 257145, were depolymerized in 3N methanolic hydrochloride (Me-OH-HCl), and cutin composition was analyzed and quantified by using GC-MS. The compositions of cutin monomers identified from the two habanero cultivars are given in Table 1. The cutin monomers from these genotypes mostly consisted of long-chain aliphatic ω-hydroxy acids, especially dihydroxy hexadecanoic acids, considered the most important component of most plant cutin materials, especially in fruits [7,24]. Parsons et al. [4] showed 16-fold differences in cutin monomer amounts between the most extreme accessions studied. Similar to this report, the metabolic analysis of cutin composition between our selected genotypes revealed significant variations in both total cutin monomer content and relative proportion of cutin. PI 257145 had the most abundant cutin content, with about 1284 mg/g dry weight (DW), and PI 224448 had the lowest cutin content, 232.4 mg/g DW. Total cutin composition and relative proportion of individual monomers varied between the two cultivars, with about 6-fold higher cutin content in PI 257145 versus PI 224448. Reports by Kissinger et al. [21] and Parsons et al. [4], showed that 10,16-dihydroxy hexadecanoic acid was the dominant cutin monomer with portions from 50% to 82% total cutin in *Capsicum annum*. Of note, our study showed a similar pattern, with PI 224448 having the lowest amount of dihydroxy hexadecanoic acid, about 114 mg/g DW (49%), and genotype PI 257145 showing the highest amount, 1060mg/g DW (83%). Among the octadecanoic acids, 9,10,12,13,18-pentahydroxy octadecanoic acid was dominant, with PI 257145 showing the highest amount, 35.3 mg/g DW. This compound was detected only in fruits, which suggests that they might play a major role in cutin composition of plants. Levels of p-coumaric acid, a phenolic compound, also showed significant variations between the two pepper genotypes. These variations between the samples provided a good background to investigate the cutin biosynthesis mechanisms by examining variations in expression of the some of the key players in this pathway.

### 2.2. Fruit Transcriptome Sequencing and Analysis

Total RNA was isolated from the green fruit tissues from the two habanero pepper genotypes, PI 257145 (high cutin) and PI 224448 (low cutin). An RNA-Seq library was prepared for each genotype separately by using total RNA pooled from three biological replicates. The library was subjected to paired-end sequencing (2 × 75 bp) with Illumina NextSeq 500 platform (Illumina, California, USA). A total of 22,550,145 and 24,056,689 reads were generated for PI 257145 and PI 224448, respectively (Table 2). The raw RNA sequencing data for the two genotypes were deposited in the Short Read Archive (SRA) database of NCBI with the accession numbers SRX6761116 and SRX6761113 for PI 257145 and PI 224448, respectively, under the bioproject PRJNA562491. The raw reads were subjected to stringent quality filtering, which resulted in 21,411,561 and 19,981,360 high-quality reads for PI 257145 and PI 224448, respectively. The Q30 percentage of reads in each library was ≥95%. The reads from the two genotypes were aligned to the *C. chinense* reference genome [25] by using the STAR universal RNA-Seq alignment tool with default parameters [26]. A total of 21,021,870 (98.18%) and 19,649,669 (98.34%) quality-filtered reads were mapped to the reference genomes for PI 257145 and PI 224448, respectively; 2% of the reads remained unmapped.

### 2.3. DEGs Between PI 257145 and PI 224448

**Figure 1:**
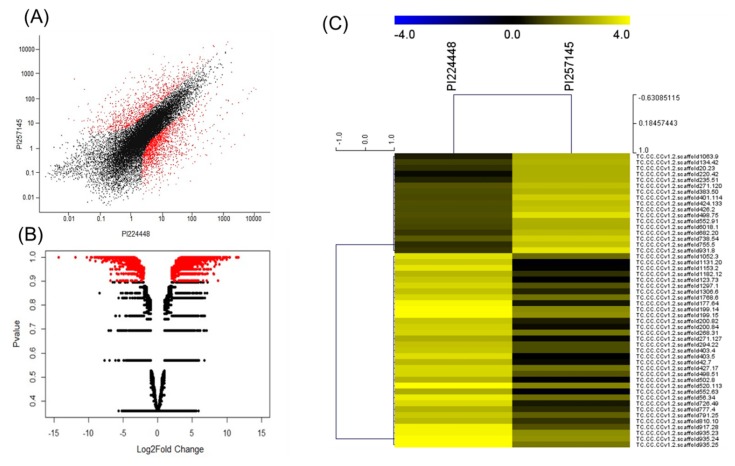
(**A**) Summary plot of expression values for the genotypes PI 257145 and PI 224448. The red points represent the genes with significant *p*-value of ≥ 0.9. (**B**) Volcano plot showing the Log2 fold change (FC) of differentially expressed genes (DEGs) in PI 257145 versus PI 224448. The Log2FC is plotted on the *x*-axis and the *p*-value is plotted on the y-axis. The red points in the scatter-plot show the DEGs with *p*-value ≥ 0.9 and the black points are less significant with *p*-value > 0.9. (**C**) Top 50 differentially expressed genes between the genotypes PI 224448 and PI 257145 based on the fragments per kilobase of transcripts per million (FPKM) normalized Log10-transformed counts. The color key yellow represents high expression and blue represents low expression.

The individual read count tables across genes for the two genotypes were created by genome alignment with the HTSeq R package and RSEM [27] with RPKM normalization. DEGs were identified by pair-wise combinations by comparing PI 257145 and PI 224448 with the use of NOISeq R/Bioc package [28] with three simulated replicates having a variability of 0.02 and CPM value 1. The DEGs were filtered based on the minimum Log2FC of 1 and *p*-value 0.9 as per the NOISeq R/Bioc package. A total of 2703 statistically significant DEGs were identified including 1693 upregulated and 1010 downregulated genes in PI 257145 versus PI 224448 (Figure 1). The top 20 upregulated genes in PI 257145 versus PI 224448 are in Table 3. The top 50 differentially expressed genes between PI 224448 and PI 257145 based on the FPKM-normalized-Log10 transformed counts are in Figure 1.

### 2.4. Functional Annotation and Classification of DEGs

**Figure 2:**
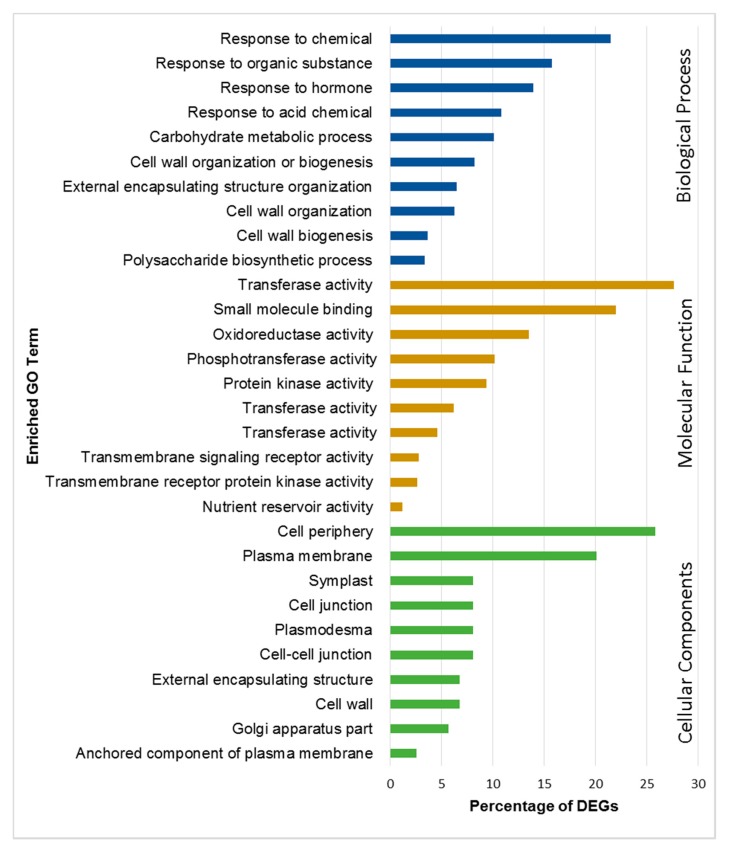
Top 10 gene ontology terms under biological process, molecular function and cellular components enriched among the DEGs upregulated in PI 257145 versus PI 224448.

DEGs were annotated by using the BLASTx algorithm and nonredundant protein database at NCBI. Gene annotation and gene ontology (GO) enrichment analysis was performed with BLAST2GO (https://www.blast2go.com/). The DEGs were classified under the three major GO terms such as biological process, molecular function and cellular components. GO classification showed significant functions of the identified DEGs in PI 257145 versus PI 224448. A total of 1071 upregulated genes in PI 257145 were classified under top ten categories of biological process. The significant categories upregulated in PI 257145 included “cell wall biogenesis”, “polysaccharide biosynthetic process”, “carbohydrate metabolic process”, “cell wall biogenesis”, “cell wall organization”, “response to hormone” and “external encapsulating structure organization”. These categories are important for the structural stability of the fruits. The molecular function category included 941 DEGs with enriched terms of “oxidoreductase activity”, “transferase activity” and “transmembrane receptor protein kinase activity”. All these enriched molecular functions are important for fruit quality and plant defense. The major cellular components enriched in DEGs upregulated in PI 257145 included “cell periphery”, “plasma membrane”, “cell wall”, “cell–cell junction”, “plasmodesma” and “external encapsulating structure”. All these cellular component terms enriched in DEGs upregulated in high-cutin PI 257145 are essentially involved in maintenance of cellular structure and fruit quality. The statistically enriched GO terms (false discovery rate (FDR) < 0.05) among the DEGs upregulated in PI 257145 versus PI 224448 are shown in Figure 2.

### 2.5. Pathway Analysis of DEGs

**Figure 3:**
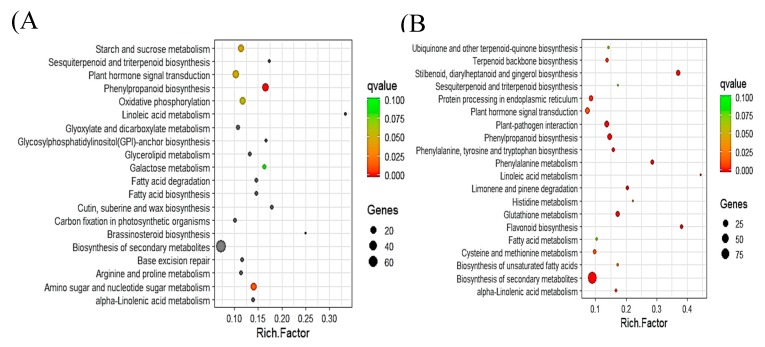
Top 20 enriched KEGG pathways among upregulated (**A**) and downregulated (**B**) DEGs in PI 257145 versus PI 224448. Rich factor is the ratio of the number of DEGs to the total gene number in a pathway. Here, q-value is a corrected *p*-value. The color and size of the dots represent the range of q-value and the number of DEGs mapped to the indicated pathways, respectively.

**Figure 4:**
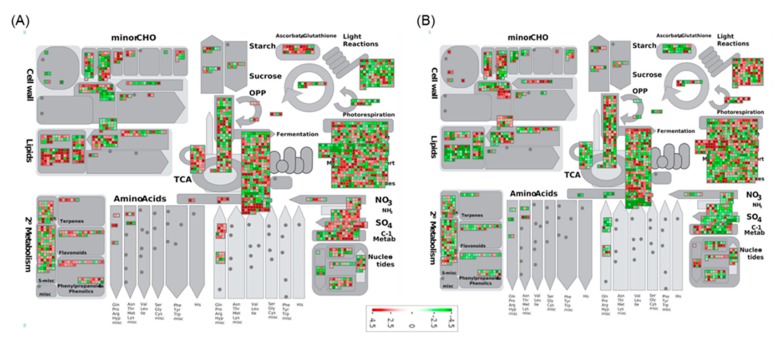
MapMan pathway analysis shows the differences in activity of different cellular metabolisms between the genotypes (**A**) PI 257145 and (**B**) PI 224448.

**Figure 5:**
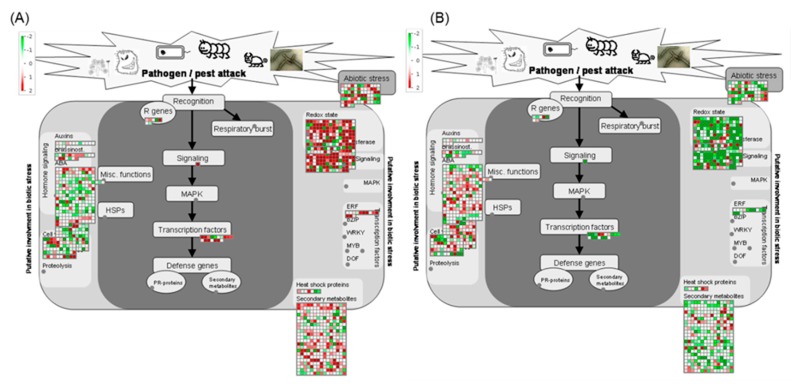
MapMan pathway analysis shows the expression of genes in biotic and abiotic stress-related pathways between the genotypes (**A**) PI 257145 and (**B**) PI 224448.

Pathway analysis of DEGs involved using the Kyoto Encyclopedia of Genes and Genomes (KEGG) pathway database with KOBAS and MapMan. The DEGs upregulated (1693) and downregulated (1010) in PI 257145 versus PI 224448 were assigned to 100 and 97 pathways, respectively. KEGG pathway analysis shown that many of the upregulated genes are enriched in pathways relevant to cutin biosynthesis and its regulation [11]. The pathways enriched in upregulated genes of high-cutin PI 257145 were “phenylpropanoid biosynthesis”, “plant hormone signal transduction”, “oxidative phosphorylation”, “biosynthesis of secondary metabolites”, “linoleic acid metabolism”, “cutin, suberine and wax biosynthesis”, “fatty acid biosynthesis”, “sesquiterpenoid and triterpenoid biosynthesis”, “alpha-linolenic acid metabolism” and “brassinosteroid biosynthesis. The top 20 enriched KEGG pathways among upregulated and downregulated DEGs in PI 257145 compared to PI 224448 are shown in Figure 3. Pathway analysis using MapMan [29] showed differences in the activity of different cellular metabolisms between PI 257145 and PI 224448. Many of the DEGs involved in lipid metabolism and secondary metabolism were highly upregulated in PI 257145 (Figure 4). Cutin composition and its genetic basis have been studied in model plants such as *Arabidopsis*, tomato and rice [14,15,16,17,23]. Studies have shown that cuticle composition varies across pepper cultivars and this variation in turn affects their responses to biotic and abiotic stresses [21,30]. The genes involved in management of biotic and abiotic stresses are highly upregulated in the high-cutin PI 257145 versus low-cutin PI 224448. The genes regulating redox state and TFs involved in regulating defense genes were upregulated in the high-cutin PI 257145 (Figure 5).

### 2.6. Functional Network Analysis of DEGs

**Figure 6:**
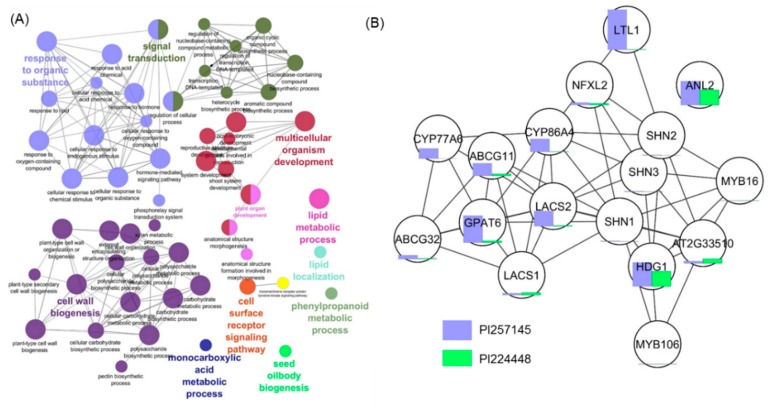
(**A**) Functional network analysis of upregulated genes in PI 257145 showing the functionally grouped terms with nodes linked based on their kappa score level (≥ 0.3), where only the label of the most significant term per group is shown. The node size represents the enrichment significance of the term. (**B**) Network analysis of genes involved in cutin biosynthesis in *Capsicum chinense*. Bar chart associated with the nodes shows the expression value fragments per kilobase of transcripts per million (FPKM) between PI 257145 and PI 224448.

Ten functional network clusters were obtained, including response to organic substance, signal transduction, multicellular organism development, cell wall biogenesis, lipid metabolic process, cell surface receptor signaling pathway, phenylpropanoid metabolic process, monocaboxylic acid metabolic process, seed oil body biosynthesis and lipid localization (Figure 6A). Most of the functional network groups were well connected to the enzymes and proteins involved in cutin biosynthesis and its regulation. Figure 6B shows the network representing an interaction between cutin genes and TFs. Bar plots are used to denote the gene expression profiles between the two genotypes and show DEGs between high- and low-cutin habanero pepper based on FPKM.

### 2.7. Analysis of DEGs for Transcription Factors (TFs)

TFs plays a major role in regulating genes for cutin biosynthesis and genes involved in biotic and abiotic stress-related pathways. TFs enriched in the DEGs were analyzed by using the Plant Transcription Factor Database (http://planttfdb.cbi.pku.edu.cn/). Among DEGs coding for TFs, 71 were upregulated and 43 were downregulated in PI 257145 versus PI 224448. The upregulated TFs represented 27 families and major TFs upregulated in PI 257145 included ERF (14), GRAS (8), MYB (4), ZF-HD (4), B3 (3), bZIP (3), C2H2 (3), C3H (3), MADS (3), NAC (3), ANL2 (3), NF-YB (2), NF-YC (2), SHN1 (2) and HDG1. Similarly, the downregulated TFs represented 16 families and major TFs downregulated in PI 257145 included ERF (13), C2H2 (7), NAC (3), WRKY (3), bHLH (2), bZIP (2), C3H (2), Dof (2), GRAS (2), HD-ZIP (2) and CFL1. Among the TF families differentially expressed, many TF families were significantly upregulated in high-cutin PI 257145. Different genes of the same TF family showed differential expression between the two genotypes. Among the TFs, 14 ERFs, 8 GRASs, 3 NACs, 3bZIPs and 3 C3Hs, were upregulated in PI 257145 and 13 ERFs, 2 GRASs, 3 NACs, 2 bZIPs and 2 C3Hs were downregulated. Furthermore, 4 MYBs, 3 B3s, 3 MADSs, 3 ANL2s, 2 SHN1s and HDG1 are uniquely upregulated in PI 257145. Among the TFs upregulated, 3 ANL2s, 2 SHN1s and HDG1 were considered important positive regulators of cutin biosynthesis [11,31,32]. These TFs playing an important role in regulating cutin biosynthesis were highly upregulated in high-cutin genotype PI 257145 versus low-cutin genotype PI 224448.

### 2.8. Genes Involved in Cutin Biosynthesis

The cutin monomers are mostly composed of long-chain aliphatic ω-hydroxy acids, especially dihydroxy hexadecanoic acids, and have been considered the most important component of most plant cutin materials, especially in fruits [7,24]. Cutin is synthesized by epidermal cells in higher plants and is an insoluble, covalently cross-linked polymer consisting of organic chemicals including glycerol, hydroxylated fatty acids and hydroxylated epoxy compounds with carbon atom chains of lengths 16 and 18 [5,12,33]. Their monomers consist of C16 or C18 aliphatic fatty acids, their derivatives and glycerol and phenolic compounds. These monomers are generated from fatty acyl-CoA by a series of hydroxylation and epoxidation reactions that are catalyzed primarily by cytochrome-P450-dependent enzymes [5,34]. Cutin polymers are essential for plant development and are synthesized via the cutin biosynthetic pathway [11]. C16/C18 fatty acid precursors are initially catalyzed by long-chain acyl-CoA synthetase (LACS) genes, and further catalysis by downstream genes yields various monomers along the cutin pathway. Several enzymes for the biosynthesis of cutin polymer have been identified in *Arabidopsis*, involving cascade of activities from long-chain acyl-CoA synthetase (LACS1/LACS2) to cutin synthase/GDSL lipase [11,35]. *Arabidopsis* homologs for genes involved in cutin biosynthesis were used to identify the corresponding homologs from *C. chinense*, and their differential expression between the two genotypes in terms of fold change were calculated (Table 4).

The CoAs are esterified to fatty acids by long-chain acyl-CoA synthetase (LACS1 and LACS2) to give acyl-CoA [35,36]. Mutations in LACS2 showed a reduced amount of cutin monomers and slight reduction in amount of cuticular wax in *Arabidopsis* [37]. LACS2 is important for the biosynthesis of cutin monomer, and in our study the expression of LACS2 was highly correlated with cutin content for the two genotypes. LACS2 was expressed three-fold higher in PI 257145 than PI 224448. We located the expression of three LACS2 genes in habanero genotypes and all three were significantly upregulated in high-cutin PI 257145 versus low-cutin PI 224448. The Log2FC values for the three LACS2 genes were 2.9, 4.2 and 3.5. Cytochrome-P450-dependent enzymes (particularly members of the CYP86A family and CYP77A) catalyze a series of hydroxylation and epoxidation reactions in epidermal cells in plants [5]. In the cutin biosynthetic pathway, CYP86A encodes a ω-hydroxylase that incorporates a hydroxyl group to give 16-hydroxy or 18-hydroxy fatty acids, whereas CYP77A carries a midchain hydroxylase or epoxidase activity for the synthesis of dihydroxy fatty acids [11,15,38]. Both enzyme families were upregulated in PI 257145 with fold-change range from 2.8 to 5.5 (Table 4).

Another enzyme in the pathway encodes the activity of an acyltransferase, glycerol-3-phospahate acyl transferase 6 (GPAT6), which adds the glycerol moieties into cutin. GPAT6 enzymes are involved in the transfer of fatty acids from acyl-CoA to glycerol-3-phosphate [11,24,39]. A gpat6-a mutant showed a striking phenotype in tomato fruit, with greatly altered cuticle thickness, composition and properties [40,41]. GPAT6 gene was expressed four-fold higher in high-cutin PI 257145 than low-cutin PI 224448. The enzyme GDSL esterase or lipase/cutin deficient 1 (CD1) encodes α-hydroxylase that is involved in the polymerization of various acyl-glycerols to give the cutin polymers. Previous reports have clearly demonstrated the role of this enzyme in cutin biosynthesis and showed a marked reduction of cutin content in GDSL lipase mutant tomato genotypes [12,42,43,44]. GDSL is considered one of the major rate-limiting enzymes for cutin biosynthesis. We have found two genes for GDSL esterase or lipase in *C. chinense*, and both were highly expressed in high-cutin PI 257145. The FC ranged from 3 to 7 in PI 257145 compared with low-cutin PI 224448. Certain ATP binding cassette (ABC) transporters, the ABCG subfamily (ABCG11 and ABCG32), have also been associated with cutin biosynthesis and are involved in the export of cutin precursors across the plasma membrane in plants [45,46,47,48]. These transporter genes are important for cutin biosynthesis. All are highly expressed in PI 257145 versus PI 224448, which agreed with the cutin content.

**Figure 7:**
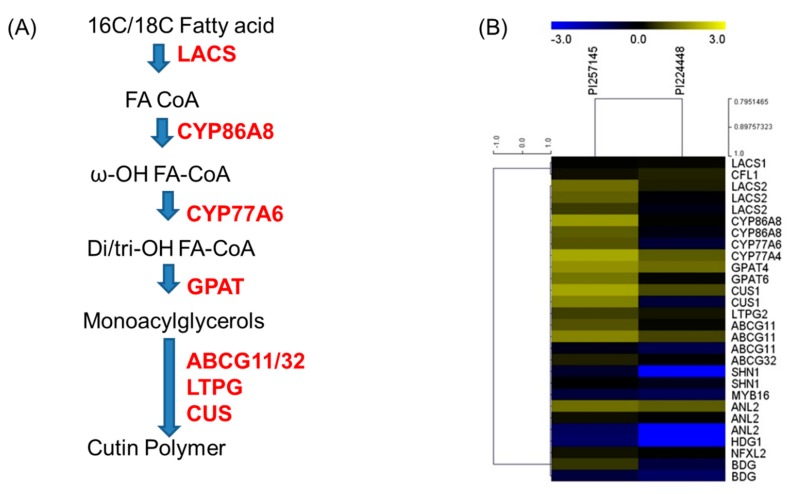
(**A**) Putative cutin biosynthetic pathway predicted in *Capsicum chinense* based on RNA-Seq data. (**B**) Expression of genes involved in cutin biosynthesis, transport, and regulation between the two genotypes PI 224448 and PI 257145 based on FPKM-normalized Log10-transformed counts. The color yellow represents high expression and blue represents low expression.

The transcriptional regulators in the cutin biosynthesis pathway play major roles in regulating biosynthetic genes. The WIN/SHN TFs were first identified in *Arabidopsis*, and there are three major SHN genes for cuticle biosynthesis (SHN1, SHN2 and SHN3) [31]. These sets of genes belong to the *Arabidopsis* APETALA 2 (AP2) family TFs and they regulate cutin and epidermal cells. WIN1/SHN1 is an activator of the promoter region of several cutin genes, and in tomato, SISHN3 has been reported to upregulate multiple genes involved in cutin metabolism, e.g., CYP86A gene of the cytochrome P450 [17,18,33,38,49]. Hence, the SHN1 TF is considered a strong positive regulator of cutin biosynthesis. Of note, SHN1 was expressed at a higher level in PI 257145 than in PI 224448, with 8-fold difference. Another set of TFs, the homeodomain leucine zipper IV (HD-Zip IV) TFs, were identified in *Arabidopsis*. They are highly expressed in epidermal cells and their functions are epidermis-related. One of these TFs, nuclear factor X-like 2 (NFXL2), has been identified as a negative repressor for all SHN genes, ultimately leading to negative alterations in cutin composition [18]. The expression of NFXL2 in PI 257145 was not significant, which agrees with high cutin content in this genotype. Another member of the class IV homeodomain–leucine-zipper proteins TFs regulating cutin biosynthesis discovered in *Arabidopsis* was anthocyaninless2 (ANL2). In [50], the leaf cutin composition in the ANL2 mutant was 40% less than in the *Arabidopsis* wild-type. Supporting this, in our study, ANL2, a positive regulator of cutin biosynthesis was expressed at higher level in PI 257145 than PI 224448, with about 6-fold difference. Overexpression of MYB30 in Arabidopsis was also reported to stimulate the synthesis of long chain fatty acids and cutin [51]. In our study, MYB protein had higher expression in high-cutin PI 257145 than low-cutin PI 224448, which further strengthens its role as a candidate regulatory factor in cutin metabolism. The putative cutin biosynthetic pathway genes predicted for habanero peppers based on RNA-Seq data are shown in Figure 7A; their expression based on FPKM-normalized Log10-transformed counts is shown in Figure 7B. Analysis of all genes for the cutin biosynthesis pathway revealed that all the genes experimentally validated to positively regulate cutin biosynthesis were significantly upregulated in high-cutin PI 257145 versus low-cutin PI 224448. The RNA-Seq based gene expression data and metabolic data showed significant correlation in cutin content and gene expression between the two habanero genotypes, which in turn identified the important genes and TFs contributing to the increased cutin content in PI 257145.

### 2.9. RNA-Seq Gene Expression Validation by RT-qPCR

**Figure 8:**
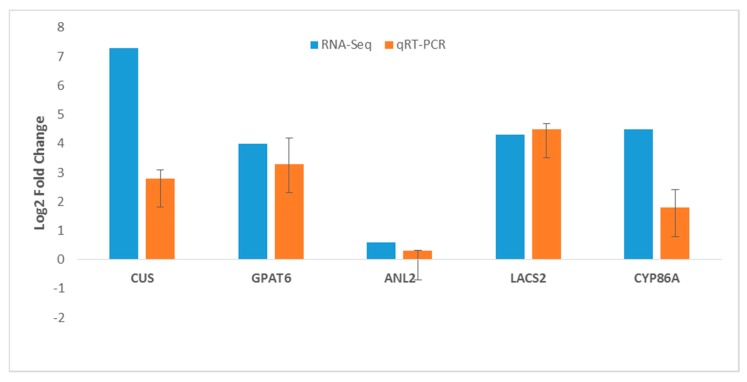
Relative gene expression of selected genes involved in cutin biosynthesis pathway by using RNA-Seq and RT-qPCR. Data represent Log2FC from high-cutin PI 257145 versus low-cutin PI 224448.

To validate the RNA-Seq data, randomly selected genes involved in the cutin biosynthetic pathway with significant expression difference between PI 257145 and PI 222448 were chosen for RT-qPCR. The selected genes were GDSL esterase/lipase (CUS), glycerol-3-phosphate 2-O-acyltransferase 6 (GPAT6), long chain acyl-CoA synthetase 2 (LACS2), HD-ZIP IV transcription factor (ANL2) and cytochrome P450 86A (CYP86A4). All the five genes were significantly upregulated in high-cutin PI 257145 versus low-cutin PI 224448. The overall results from RT-qPCR were consistent with RNA-Seq data (Figure 8).

Integrating metabolomic and transcriptomic analysis revealed significant differences in cutin biosynthesis between the habanero genotypes PI 257145 and PI 224448. Metabolomics analysis revealed about 6-fold higher cutin content in PI 257145 versus PI 224448. Transcriptomic analysis revealed several significant DEGs between the high- and low-cutin genotypes. Genes such as GDSL lipase, glycerol-3 phosphate acyltransferase 6, long-chain acyltransferase 2 and cytochrome P450 86A/77A were found to be important for cutin biosynthesis. TFs such as SHN1, ANL2 and HDG1 are found to be the key regulators of the cutin biosynthetic pathway.

## 3. Materials and Methods

### 3.1. Collection of Plant Material

Seeds from two different habanero pepper genotypes (PI 224448 from Costa Rica and PI 257145 from Peru) from a worldwide collection of habanero peppers were obtained from USDA GRIN. Ten plants for each line were started in the greenhouse as surface-sterilized seeds in pots. The seeds were sown in moderately wet soil and covered with black paper bags in the dark for about 3 to 4 days to germinate in a temperature- and humidity-controlled incubator. After 4 days of germination in darkness, the pots were removed from the incubator, uncovered and left to grow under controlled conditions in the greenhouse, watered daily and finally transplanted to the Sissonville field plots. The plants were allowed to mature, and the appearance of waxes or glossiness guided our selection for the fruit sample collection. Mature green fruit tissues from each of the genotypes flowered at the same time were collected, frozen in liquid nitrogen and stored at −80 °C.

### 3.2. Cutin Isolation and GC-MS Analysis

Detailed metabolite profiling involved GC-MS. Cutin composition of the fruit tissues of the genotypes were examined with three replications according to the protocol reported by Parsons et al. [4] with slight modifications. Cuticle was isolated from 50 mg frozen fruit tissue powder obtained from lyophilized matured green fruits. Enzymatic digestion of the powdered samples involved using 2% pectinase and 0.1% cellulase in 0.2 mM citrate buffer, 3.7 pH (using 0.001% phenylmercuric nitrate as an antimicrobial agent). An incubator–shaker was set at 35 °C and 100 rpm for several days until the discs had little or no debris on them. Acetone with 50 mg L^−1^ butylated hydroxytoluene was used to rinse the isolated cuticles three times, followed by refluxing delipidation of the discs in chloroform:methanol (1:1, *v*/*v*). Depolymerization in 3N methanolic hydrochloride (Me-OH-HCl) was then performed by using a protocol by [52] with 6.5 mL of 3 N Me-OH-HCl for each depolymerization reaction and left for 16 h at 60 °C. The reaction vials were cooled to room temperature, and 6 mL saturated aqueous NaCl was added to stop the depolymerization reaction. The individual cutin monomers were removed as methyl esters in two different extractions by using distilled dichloromethane [53] Centrifugation at 3000 rpm for 3 min was used to separate the different phases, followed by washing the organic phase with 0.9% aqueous NaCl three times and incubation with 2,2-dimethoxy propane at 60 °C to remove dissolved water in the organic phase and then drying under nitrogen gas. BSTFA was used for derivatization followed by GC-FID analysis as previously described [54]. An Agilent 5975C GC-MS instrument with an HP-5 MS column (30 m, 0.25 mmID, 0.25 µm film) was used, and methyl heptadecanoate and methyl tricosanoate were used as internal standards. Published mass spectra of methyl ester and trimethyl silyl derivatives were used to identify the monomers ([55]; http://lipidlibrary.aocs.org/). The amount of individual cutin monomers was expressed in milligrams/gram dry weight.

### 3.3. RNA Isolation, Library Preparation and Transcriptome Sequencing

Total RNA was isolated from 100 mg matured green fruit tissues of the two genotypes PI 257145 and PI 224448 with three biological replicates by using the Nucleospin RNA plant kit (Macherey Nagel). Total RNA was treated with DNAseI (Qiagen) to remove co-isolated genomic DNA and purified by using the RNeasy MinElute Cleanup Kit (Qiagen). The Qubit 4 Fluorometer (Invitrogen) and Agilent 2100 Bioanalyzer were used to detect the concentration and integrity of total RNA. Total RNA from three replicates was pooled for each genotype before RNA-Seq library preparation. Libraries for the RNA-Seq of the two habanero genotypes were prepared by using the NEBNext Ultra II RNA Library Prep Kit according to the manufacturer’s specification. Taking 1 µg total RNA, mRNA enrichment for poly-A involved using magnetic beads with Oligo (dT) with NEBNext Poly (A) mRNA Magnetic Isolation Module (NEB, E7490) followed by fragmentation into shorter fragments by using fragmentation buffer. Oligo dT primers were used for synthesis of first-strand cDNA. Sequencing adapters were added to the resulting cDNA followed by amplification of the library using sequencing primers. After constructing the RNA-seq library, the Agilent 2100 Bioanalyzer (Invitrogen) was used to analyze the library insert size, and the Qubit 4 Fluorometer (Invitrogen) was used to quantify the library concentration. RNA-Seq for each of the samples involved using the Illumina NextSeq 500 platform with a paired-end sequencing protocol. The resulting image files in the bcl format were converted to FASTQ with 2 × 75 bp reads with the bcl2fastq tool (Illumina).

### 3.4. Transcriptome Analysis

The quality of raw reads was ascertained by checking the adapter, GC distribution, average base content and quality score of the distribution by using fastqc (https://www.bioinformatics.babraham.ac.uk/projects/fastqc/). The adapter sequences and low-quality reads (Phred score QV < 30) were removed and the clean reads were filtered from the raw data by using the software cutadapt (https://cutadapt.readthedocs.io/en/stable/guide.html) and sickle (https://github.com/najoshi/sickle), respectively. The quality-filtered reads were mapped to the *C. chinense* reference genome v1.2 (http://peppergenome.snu.ac.kr/) by using the STAR universal RNA-Seq alignment tool with default parameters [26] to generate BAM alignment. The read count tables for the genes across all the samples were created by using BAM alignment and the general feature format (GFF) of genome annotation with the HTSeq R package [27] and RSEM (https://deweylab.github.io/RSEM/). The counts were normalized by using reads per kilobase of transcripts per million (RPKM). The gene expression based on the read counts were studied using fragments per kilobase of transcripts per million (FPKM). The FPKM values for each of the genes were calculated based on the read count table, the total number of reads per sample and gene length in kb.

The DEGs resulting from the comparison of PI 257145 and PI 224448 were identified using the NOISeq R/Bioc package [28] with three simulated replicates having variability of 0.02 and counts per million (CPM) of 1. The DEGs were filtered based on the minimum Log2FC of 1 and *p*-value of 0.9 as per the NOISeq R/Bioc package. Gene annotation, gene ontology (GO) enrichment analysis was performed with BLAST2GO (https://www.blast2go.com/). Transcription factor (TF) prediction, and TF enrichment analysis was involved using the Plant Transcription Factor Database (http://planttfdb.cbi.pku.edu.cn/). Heatmaps were generated by using mev (http://mev.tm4.org/). Gene network analysis involved using Cytoscape (https://cytoscape.org/) and the STRING database (https://string-db.org/) with *Arabidopsis* as the reference to retrieve protein–protein interactions. Functional networks for DEGs were derived by using the ClueGO plugin [56] available in Cytoscape. Pathway mapping involved using KOBAS [57] and MapMan (https://mapman.gabipd.org/). Sequences of genes involved in cutin biosynthesis were identified by using the Arabidopsis homolog and *C. chinense* mRNA sequences [11].

### 3.5. RT-Quantitative PCR (RT-qPCR)

Total RNA was isolated from frozen matured green fruit tissues of habanero pepper by using the Plant RNA mini spin kit (Macherey-Nagel). The NanoDrop 2000 Spectrophotometer (Thermo Fisher Scientific, MA, USA) was used to measure RNA concentrations. The Super Script First-Strand Synthesis system (Invitrogen) was used for first-strand cDNA synthesis with 6 µg total RNA per sample. An amount of 1 µL cDNA diluted 1:6 was used for RT-qPCR analysis. In a final volume of 20 µL, diluted cDNA was mixed with 10 µL SYBR Green PCR master mix (Applied Biosystems, Foster City, CA, USA) and 10 pmol each of forward and reverse primers and completed with nuclease free water. Primer3Plus software (http://www.primer3plus.com/) was used to design gene-specific primers for the randomly selected genes involved in cutin biosynthesis. Details of the genes with primer sequences are available in Supplementary Table S1. Semiquantitative RT-PCR amplification to test primers was performed in a total reaction volume of 20 µL containing 1 µL cDNA, 10 µL colorless GoTaq and 10 pmol each of forward and reverse primers and completed with nuclease free water. Thermocycling conditions were an initial denaturing step of 95 °C for 1 min, followed by 25 cycles of 95 °C for 15 s, corresponding annealing temperature 60 °C for 70 s and 72 °C for 30 s, with a final extension step of 72 °C for 25 min. An amount of 1% agarose gel pre-stained with ethidium bromide was used to confirm the amplified fragments by visualization under UV light. Transcript-level expression was detected by RT-qPCR with SYBR Green PCR Master mix (ROX) (Roche, Shanghai) on a StepOnePlus Real-Time PCR System (Applied Biosystems, Foster City, USA). PCR involved a total reaction volume of 20 µL containing 1 µL cDNA, 1 µL of the forward and reverse primers (10 µM), 10 µL of SYBR Green PCR Master mix (ROX) (Roche, Shanghai, China) and 8 µL sterile distilled water. Amplification conditions were 95 °C for 10 min, followed by 40 cycles of 95 °C for 15 s, and 60 °C for 1 min. The reactions were performed in three technical replications and three biological replicates to compute the average Ct values. The gene expression for each gene was normalized against beta-tubulin expression and data analysis for the relative gene expression was computed with the 2^-∆∆*C*T^ method. The results are expressed as Log2foldchange (Log2FC) ± mean standard error (SEM).

## 4. Conclusions

Integrating metabolomic and transcriptomic analysis revealed significant differences in cutin biosynthesis between the habanero genotypes PI 257145 and PI 224448. Metabolomics analysis revealed significant variations in cutin composition between the two genotypes, with about 6-fold higher cutin content in PI 257145 versus PI 224448. Cutin monomer 10,16-dihydroxy hexadecanoic acid was present at the highest percentage (82.6%) in PI 257145. Transcriptomic analysis with RNA-Seq revealed significant gene expression differences between the high- and low-cutin genotypes. In this study, we report transcriptome and metabolome data pertaining to cutin in habanero peppers along with the predicted putative cutin biosynthetic pathway for habanero peppers. Genes such as GDSL lipase, glycerol-3 phosphate acyltransferase 6, long-chain acyltransferase 2 and cytochrome P450 86A/77A and TFs such as SHN1, ANL2 and HDG1 are found to be the key genes highly contributing to the high cutin content in PI 257145. These genes previously showed a similar pattern of regulation in tomato and *Arabidopsis*. These analyses advance our knowledge on the molecular mechanisms regulating the accumulation of cutin in habanero pepper fruits. These resources can be built on for developing habanero fruit cultivars with high cutin content that show resistance to biotic and abiotic stresses.

## Figures and Tables

**Table 1:** Cutin monomers identified from habanero pepper fruits quantified by GC-MS.

Cutin Monomers	PI 224448		PI 257145	
	Mean ± SD	%	Mean ± SD	%
Hexadecanoic acid	11.2 ± 2.6	4.8	16.1 ± 6.0	1.3
10,16-Dihydroxy hexadecanoic acid	114.1 ± 19.7	49.1	1060.1 ± 495.4	82.6
16-Hydroxy hexadecanoic acid	39.7 ± 15.8	17.1	77.3 ± 11.4	6.0
Octadecanoic acid	4.7 ± 0.8	2.0	6.3 ± 2.1	0.5
9,10,12,13,18-Pentahydroxy octadecanoic acid	18.1 ± 4.4	7.8	35.3 ± 11.5	2.8
9,10,18-Trihydroxy octadecanoic acid	2.1 ± 0.8	0.9	2.8 ± 1.8	0.2
Octadecenoic acid	0.6 ± 0.4	0.3	4.1 ± 0.3	0.3
Octadec-9-enoic acid	0.6 ± 0.3	0.2	0.7 ± 0.4	0.1
18-Hydroxy octadecenoic acid	1.4 ± 0.5	0.6	5.3 ± 1.0	0.4
Octadecadienoic acid	5.0 ± 2.3	2.2	14.8 ± 1.4	1.2
18-Hydroxy octadecadienoic acid	6.0 ± 2.6	2.6	7.1 ± 2.4	0.6
p-Coumaric acid	28.7 ± 7.6	12.3	54.0 ± 37.4	4.2
Total cutin	232.4 ± 57.8	100.0	1284.0 ± 571.0	100.0

Data are mean ± standard deviation mg/g dry weight from three independent biological replications.

**Table 2:** Summary of RNA-Seq and reference genome alignment in fruit tissue of *Capsicum chinense* Jacq.

Particulars	PI 257145	PI 224448
Total raw reads	22,550,145	24,056,689
Total valid paired-end reads	21,411,561	19,981,360
Read length	75	75
GC content (%)	41	43
Q30 (%)	95.2	95.7
Mapped reads	21,021,870(98.18%)	19,649,669 (98.34%)
Unmapped reads	389,691 (1.82%)	331,691 (1.66%)
Unique mapped reads	19,802,144 (92.48%)	17,391,436 (87.03%)
Multiple mapped reads	1,100,712 (5.14%)	1,569,061 (7.85%)

**Table 3:** Top 20 upregulated genes in PI 257145 versus PI 224448.

Name	Annotation	Log2FC	PI 257145 (FPKM)	PI 224448 (FPKM)
TC.CC.CCv1.2.scaffold1153.2	Glycine-rich protein-like	11.68	4066.11	1.24
TC.CC.CCv1.2.scaffold403.5	BURP domain protein USPL1-like	11.59	3119.46	1.01
TC.CC.CCv1.2.scaffold917.27	Uncharacterized mitochondrial protein AtMg00810-like	11.58	141.33	0.05
TC.CC.CCv1.2.scaffold543.19	Nonspecific lipid-transfer protein A-like	11.57	655.87	0.22
TC.CC.CCv1.2.scaffold177.64	Wound-induced protein	11.50	8286.74	2.85
TC.CC.CCv1.2.scaffold1131.20	Protein EXORDIUM-like 2	11.36	1497.42	0.57
TC.CC.CCv1.2.scaffold260.25	Probable cellulose synthase A catalytic subunit 3]	10.41	47.52	0.04
TC.CC.CCv1.2.scaffold123.73	Proteinase inhibitor PSI-1.2-like	10.34	1359.44	1.05
TC.CC.CCv1.2.scaffold552.63	Proline-rich receptor-like protein kinase PERK13	10.02	488.72	0.47
TC.CC.CCv1.2.scaffold327.13	Haloacid dehalogenase-like hydrolase domain-containing protein 3	9.99	13.39	0.01
TC.CC.CCv1.2.scaffold217.2	Patatin group D-3-like	9.87	57.32	0.06
TC.CC.CCv1.2.scaffold543.15	Nonspecific lipid-transfer protein A-like	9.79	442.52	0.00
TC.CC.CCv1.2.scaffold200.84	Proline-rich extensin-like protein EPR1	9.73	1784.23	2.10
TC.CC.CCv1.2.scaffold726.49	Em protein H5	9.69	2630.41	3.17
TC.CC.CCv1.2.scaffold1580.5	Probable polyamine oxidase 4	9.66	26.49	0.03
TC.CC.CCv1.2.scaffold260.9	Chlorophyll a-b binding protein 3C, chloroplastic	9.56	198.21	0.26
TC.CC.CCv1.2.scaffold223.6	GDSL esterase/lipase At4g01130-like	9.55	168.16	0.22
TC.CC.CCv1.2.scaffold600.23	NADPH-dependent aldehyde reductase 1, chloroplastic-like	9.41	301.84	0.44
TC.CC.CCv1.2.scaffold323.10	Neutral ceramidase-like	9.38	13.37	0.02
TC.CC.CCv1.2.scaffold161.6	Zinc finger CCCH domain-containing protein 32-like isoform X1	9.13	14.35	0.03

FC, fold change; FPKM, fragments per kilobase of transcripts per million.

**Table 4:** Expression of genes and transcriptions factors involved in cutin biosynthesis identified from *Capsicum chinense* genotypes.

SeqName	Gene Name	Annotation	Function	Arabidopsis Ortholog	PI 257145 (FPKM)	PI 224448 (FPKM)	Fold Change (FC)	Log2FC
**Biosynthesis**								
TC.CC.CCv1.2.scaffold339.9	*LACS1*	Long chain acyl-CoA synthetase 1	Attachment of CoA to free fatty acids	AT2G47240	1.21	1.532	0.78982	−0.3404
TC.CC.CCv1.2.scaffold383.59	*LACS2*	Long chain acyl-CoA synthetase 2		AT1G49430	19.408	2.595	7.479	2.90285
TC.CC.CCv1.2.scaffold383.57	*LACS2*	Long chain acyl-CoA synthetase 2		AT1G49430	14.165	0.722	19.6191	4.29419
TC.CC.CCv1.2.scaffold383.60	*LACS2*	Long chain acyl-CoA synthetase 2		AT1G49430	5.567	0.484	11.5021	3.52382
TC.CC.CCv1.2.scaffold449.40	*CYP86A8*	Cytochrome P450 86A	ω-Hydroxylase	AT2G45970	61.423	1.179	52.0975	5.70314
TC.CC.CCv1.2.scaffold419.19	*CYP86A8*	Cytochrome P450 86A		AT2G45970	12.48	0.547	22.8154	4.51193
TC.CC.CCv1.2.scaffold1130.1	*CYP77A6*	Cytochrome P450 77A	Midchain hydroxylase	AT3G10570	10.376	0.225	46.1156	5.52718
TC.CC.CCv1.2.scaffold159.143	*CYP77A4*	Cytochrome P450 77A	Epoxidase	AT5G04660	90.595	12.342	7.34038	2.87586
TC.CC.CCv1.2.scaffold419.22	*GPAT4*	Glycerol-3-phosphate 2-O-acyltransferase 4	Synthesis of 2-monoacylglycerols	AT1G01610	50.493	18.174	2.77831	1.47421
TC.CC.CCv1.2.scaffold29.10	*GPAT6*	Glycerol-3-phosphate 2-O-acyltransferase 6		AT2G38110	23.967	1.422	16.8544	4.07506
TC.CC.CCv1.2.scaffold387.10	*CUS1*	GDSL esterase/lipase	Polymerization of 2-monoacylglycerols monomers	AT3G04290	84.079	7.607	11.0528	3.46635
TC.CC.CCv1.2.scaffold120.8	*CUS1*	GDSL esterase/lipase		AT3G04290	32.9	0.203	162.069	7.34046
**Transport**								
TC.CC.CCv1.2.scaffold236.42	*LTPG2*	Lipid transfer protein	Transport of lipids through the cell wall	AT3G43720	5.923	2.107	2.81111	1.49114
TC.CC.CCv1.2.scaffold810.2	*ABCG11*	ABC transporter G family member 11	Export of monoacylglycerols	AT1G17840	10.092	1.494	6.75502	2.75596
TC.CC.CCv1.2.scaffold791.2	*ABCG11*	ABC transporter G family member 11		AT1G17840	36.434	6.913	5.27036	2.3979
TC.CC.CCv1.2.scaffold814.31	*ABCG11*	ABC transporter G family member 11		AT1G17840	0.457	0.134	3.41045	1.76996
TC.CC.CCv1.2.scaffold877.25	*ABCG32*	ABC transporter G family member 32		AT2G26910	2.759	0.746	3.69839	1.8869
**Regulation**								
TC.CC.CCv1.2.scaffold498.34	*SHN1*	AP2 transcription factor	Positive regulator	AT1G15360	0.275	0.001	275	8.10329
TC.CC.CCv1.2.scaffold772.31	*SHN1*	AP2 transcription factor		AT1G15360	1.043	0.399	2.61404	1.38628
TC.CC.CCv1.2.scaffold680.25	*MYB16*	MYB transcription factor		AT5G15310	0.191	0.108	1.76852	0.82254
TC.CC.CCv1.2.scaffold101.83	*ANL2*	HD-ZIP IV transcription factor		AT4G00730	20.477	12.96	1.58002	0.65994
TC.CC.CCv1.2.scaffold191.43	*ANL2*	HD-ZIP IV transcription factor		AT4G00730	1.863	0.731	2.54856	1.34968
TC.CC.CCv1.2.scaffold449.31	*ANL2*	HD-ZIP IV transcription factor		AT4G00730	0.087	0.001	87	6.44294
TC.CC.CCv1.2.scaffold449.30	*HDG1*	HD-ZIP IV transcription factor		AT3G61150	0.068	0.001	68	6.08746
TC.CC.CCv1.2.scaffold23.22	*NFXL2*	Zinc-finger transcription factor	Negative regulator	AT5G05660	1.998	1.094	1.82633	0.86894
TC.CC.CCv1.2.scaffold662.10	*CFL1*	WW domain protein		AT2G33510	1.859	2.924	0.63577	-0.6534
TC.CC.CCv1.2.scaffold1560.12	*BDG*	α/β-Hydrolase	Unknown	AT1G64670	4.569	0.17	26.8765	4.74827
TC.CC.CCv1.2.scaffold366.17	*BDG*	BAHD acyltransferase		AT1G64670	0.174	0.072	2.41667	1.27302

## Supplementary Materials

Supplementary materials can be found at https://www.mdpi.com/1422-0067/21/4/1397/s1.
