# Supplementary material for: Integrated Metabolomic and Transcriptomic Analysis to Characterize Cutin Biosynthesis between Low- and High-Cutin Genotypes of Capsicum chinense Jacq
Source: Int J Mol Sci. 2020 Feb 19;21(4):1397. doi: 10.3390/ijms21041397 (PMC7073079; doi:10.3390/ijms21041397)
Supplement: Supplementary file 1 [file ijms-21-01397-s001.pdf]

Table S1. Primers of selected genes used for RT-qPCR analysis.

Gene ID	Sequence (5'->3')	Amplicon length(bp)
TC.CC.CCv1.2.scaffold120.8	F:CTCTCCGTGGGGTGAAATGG	114
	R: ATGCTGTGGACAAGGACCA	
TC.CC.CCv1.2.scaffold29.10	F: ATAGCCGATTTTCCGCCGTT	134
	R: GGGACTACTGTCCGTGGGTA	
TC.CC.CCv1.2.scaffold101.83	F: TACGCGCCAGTTGATATCCC	127
	R: CAGCACTTGACCCACGAGAT	
TC.CC.CCv1.2.scaffold383.57	F:AAGCCTCGGAGTTGGTGTTT	142
	R:CCGTGGCAGTTCCAGAAAGA	
TC.CC.CCv1.2.scaffold419.19	F: TCCAGCTGTACAAGGCTTCG	134
